# Medical History from the Earliest Times

**Published:** 1893-06-17

**Authors:** E. T. Withington


### Medical History from the Earliest Times.

By E. T. Withington, M.A., M.B. Oxon.

LVIII.—THE CLINICIANS (*continued*).

Hermann Boerhaave, though born only a few months after Baglivi, survived him thirty years, during most of which he held an unrivalled position as the medical teacher of all Europe. The beauty of his personal character which made him the idol of his pupils, the number of the latter which necessitated pulling down the walls of Leyden to make room for them, his fame which spread from China to Peru, and his receipt of a letter from a Celestial correspondent, addressed to "Boerhaave in Europe," are among the common places of medical history. But we must here confine ourselves to a short account of his teaching, and its influence upon the progress of the healing art. Boerhaave's "Aphorism" and "Institutes," once the universal test-books of medicine, are now utterly forgotten, and the cause of their former success must be sought, not in any new and important discoveries or theories they contain, but in the prestige of the writer's name, in the clearness and simplicity with which they are written, and in the elcectic character of their teaching, in which the doctrines of the mechanical school are mingled with the "solidar pathology" of Baglivi, together with smaller proportions of the chemical and humoral theories. According to Boerhaave the body is composed of solids in the shape of "fibres" and vessels, and of fluids contained in the latter, and acted upon by them according to the laws of hydrostatics. Health consists in the normal action and reaction between the solids and fluids; in disease the fibres may either be relaxed, giving rise to passive congestions, or constricted, so as to produce obstruction and suppression of secretions, while in the other cases there may be an abnormal acridity of the humours, or their circulation may be impeded by an excessive viscosity. Respiration, he considers, is a purely physical process, the pressure of the air in the pulmonary vesicles serving to complete the mixture of the blood with the chyle, and so to preserve its proper fluidity. He is puzzled, however, by the fact that death occurs if the air is not continually renewed, and asks, "Does the elastic part of the atmosphere get absorbed, or is it the hidden aliment of life as the alchemists suppose? This is a very difficult question." One of Boerhaave's chief services to medicine was his emphasis of its positive or scientific side, in contrast to the abstract philosophic theories which so abounded in the eighteenth century. "Every vital action (he says) depends on certain bodily conditions and relations; every change in these bodily conditions and relations is necessarily followed by a corresponding change in the vital activity; medicine, therefore, must be based upon physiology." This doctrine was further developed by his illustrious pupil Von Haller, and afterwards by Bichat, and produced, as we shall see, most important results. Equally valuable was his clinical teaching, in which he carried on and extended the work of Sylvius, and which was afterwards transplanted to a wider theatre by his pupils, Van Swieten and De Haen, the founders of the so-called "first Vienna school." Unfortunately, only his more theoretical lectures have survived, and those in very imperfect and unsatisfactory versions; but Haller has collected and edited a few "Consultations," which give us a somewhat better idea both of his clinical skill and of his never-failing kindliness and courtesy. The following is the first of these, slightly condensed:—

Letter from the local practitioner. "Your aid, Vir spectatissime, is implored by Mrs. W——, aged 31, of a family very liable to phthisis. Her extreme thinness, general prostration, feet swollen in the evening, &c., clearly show that the fibres are too relaxed. Till her twentieth year she enjoyed good health, when an acute fever gave rise to chlorosis, and all that heap of hysteric symptoms to which poor women are liable, especially headache and mental depression. Over-sedulous doctors did not hesitate to treat this by large venesections, at least six times repeated. Hence she got a cough, which has increased during the last seven years, and which she has innocently but imprudently encouraged by late hours and nocturnal dances. She is not gluttonous nor addicted to spirituous liquors. She complains of occasional constriction of the chest with slight pain. There is a little expectoration with the cough, increasing on suppression of the natural perspiration. She has had one or two hectic flushes, and is gradually losing flesh. She married at twenty-six, and had a daughter three years later. During pregnancy her face was covered with blotches which have not yet entirely disappeared. Adieu, most learned sir, and humanely impart the method of treatment you consider most promising, which will doubtless result in the benefit of this poor lady, and the increase of your own reputation."

Boerhaave's reply. "I have considered this case, which has been wisely observed, exactly described, and hitherto prudently treated by you. I am compelled to think that the cause of the evil must be sought in the excessive feebleness of her bodily structure, and especially in a weakness of the pulmonary tissue. Wherefore, I consider that all evacuative and debilitating treatment should be avoided. I earnestly advise the use of tonics, friction of the body, suitable exercise, nutritious and easily digested food, &c., and I add these few medicines. Let her take three of the pills A every three hours, and then one ounce of the medicated wine B, if possible, on an empty stomach. I would suggest that this treatment, if it commends itself to your judgment, be continued for two months; but use your own authority, excellent and learned sir, for correcting this, for you are fully competent. Condescend to inscribe me on your list of friends. Farewell. A.—Assafœtida, half a drachm; catechu, martech, obtanum, balsam of Peru, extract of liquorice, each one drachm; divide into pills of three grains each. B.—Iron filings, sandalwood and various aromatics, in French or Portugese wine."

The surgery of the 17th century is much less important than that which came before or after it, for the wonderful progress of physiology seems to have attracted the ablest minds to the study of medical problems, just as the anatomical discoveries of the preceeding age gave birth to the great surgeons whose work has already been considered. Still, there are a few names which cannot be entirely omitted. Richard Wiseman, though scarcely an Ambrose Paré, is in some sense the father of English surgery, but his life and work have been so recently and so fully discussed by distinguished members of our profession that we may here dismiss him with Haller's estimate, "A man of much experience, sincere, and not ashamed to confess his mistakes, not careful about the subtleties of the art, rather too fond of medicaments, and too sparing of operations."

The name of Scultetus of Ulm (1595-1645), who distinguished himself by his ingenuity in devising artificial limbs, eyes, noses, and surgical instruments, is still remembered by a bandage which he invented; and he has left us several "observations" which throw light upon the practice of his time. He was a bold operator, even in his student days: "While I was studying medicine at Padua, a noble undergraduate suffered for some months from a swelling of his left hand, which was not benefited by general or local treatment, and began to ulcerate in the palm. So we consulted the illustrious Spigelius, who, putting a probe in the ulcer, reached carious bone, and declared it was "spina ventosa," an incurable disease, due to some humour which attacks bones first and corrodes them without affecting the periosteum or causing much pain; then it forms a slightly painful swelling, and after some months the part ulcerates. I got permission of the patient, and having amputated his hand below the carpus, found the metacarpal bones corroded, but still covered with periosteum, except where the ulcer was."

The figure of Nicholas Tulp (1595-1678) is widely known from Rembrandt's famous picture, "The Anatomists." He holds an honourable place in general history as the aged Burgomaster whose intrepid patriotism prevented the surrender of Amsterdam to the French, 1672; and he deserves mention here as a skilful surgeon, whose small volume of "Medical Observation" has been called by Haller a "golden work" (aureum opus). The following are extracts therefrom: "A distinguished painter, troubled by melancholia, thought that all his bones were soft like wax, and that his body would roll up in a ball if he moved, so he stayed in bed for a whole winter. I determined not to contradict his delusion, but to use a stratagem, and told him the disease was well known to physicians, and could easily be cured, so that if he took the medicines prescribed his bones would become hard in three days and he would walk in a week. Then I gave him some simple laxative, and on the third day allowed him to sit up, but by no means to walk, doing it gradually that he might not suspect the stratagem. By the sixth day he was perfectly well; he overwhelmed me with gratitude, and ever after had the greatest confidence in medicine, for he never found out the trick, though by no means a fool in other matters, and hardly second to any in his art."

On another page he tells the story of a blacksmith who performed the operation of perinæal lithotomy on himself, and successfully extracted a stone weighing four ounces, and larger than a hen's egg, with no other instruments than a common knife and his fingers; a deed which, says Tulp, may compare with the most valiant acts recorded in history. Tulp strongly disapproves of the increasing habit of writing medical books in the vulgar tongue instead of in Latin. It will, he thinks, cause a marked increase both of real and imaginary diseases, and as an example of this he relates how a patient of his own, who had fractured and dislocated his fibula, got hold of the works of Ambrose Paré, and applied his description of the various disasters which may follow a fracture of the thigh bone to his own case. By perpetual anxiety and brooding thereupon he became sleepless, and finally went out of his mind.

